# DPIE [2-(1,2-diphenyl-1H-indol-3-yl)ethanamine] Augments Pro-Inflammatory Cytokine Production in IL-1β-Stimulated Primary Human Oral Cells

**DOI:** 10.3390/ijms19071835

**Published:** 2018-06-22

**Authors:** Sun-Hee Ahn, Jin-Kyung Lee, Nam Doo Kim, Seok-Ho Kim, Sunwoo Lee, Seunggon Jung, Kee-Oh Chay, Tae-Hoon Lee

**Affiliations:** 1 Department of Oral Biochemistry, Dental Science Research Institute, School of Dentistry, Chonnam National University, Gwangju 61186, Korea; sun3193@jnu.ac.kr; 2 Department of Molecular Medicine (BK21plus), Chonnam National University Graduate School, Gwangju 61186, Korea; wlsrud1945@naver.com; 3 NDBio Therapeutics Inc., S24 Floor, Songdogwahak-ro 32, Yeonsu-gu, Incheon 21984, Korea; namdoo@ndbio.co.kr; 4 Aging Research Center, Korea Research Institute of Bioscience and Biotechnology (KRIBB), Daejeon 34141, Korea; kims@kribb.re.kr; 5 Department of Chemistry, Chonnam National University, Gwangju 61186, Korea; sunwoo@chonnam.ac.kr; 6 Department of Oral and Maxillofacial Surgery, School of Dentistry, Chonnam National University, Gwangju 61186, Korea; ashgray79@gmail.com; 7 Department of Biochemistry, Chonnam National University Medical School, Gwangju 61469, Korea; kochay@jnu.ac.kr

**Keywords:** oral fibroblasts, IL-1β, IL-1R1, SPR, pro-inflammatory cytokines

## Abstract

Interleukin-1β (IL-1β) is a prominent pro-inflammatory cytokine that is implicated in a variety of autoimmune diseases and plays an important role in host defense against infections. IL-1β activity increases with its increasing binding capacity to IL-1 receptors (IL-1Rs). Thus, numerous studies have targeted the discovery of molecules modulating the interactions between IL-1β and IL-1R1. We have conducted an IL-1R1 structure-based virtual screening to identify small molecules that could alter IL-1β activity, using in silico computational analysis. Sixty compounds from commercial libraries were predicted to bind to IL-1R1, and their influence on cytokine production in IL-1β-stimulated gingival fibroblasts (GFs) was determined. Of these, only (2-(1,2-diphenyl-1H-indol-3-yl)ethanamine (DPIE) showed a synergistic increase in inflammatory molecules and cytokine production (IL-6, IL-8, and COX-2) at both mRNA and protein levels in IL-1β-stimulated GFs. The enhancing activity of DPIE in IL-1β-induced cytokine production increased in a dose-dependent manner without cytotoxicity. This pattern was also observed in IL-1β-stimulated primary human periodontal ligament cells (PDLs). Furthermore, we measured the impact of DPIE on the IL-1β–IL-1R1 system using surface plasmon resonance and demonstrated that DPIE increased the binding affinity of IL-1β to IL-1R1. These data indicate that DPIE boosts IL-1β signaling by enhancing the binding of IL-1β to IL-1R1 in oral primary cells.

## 1. Introduction

Periodontal diseases are chronic inflammatory conditions caused by an inflammatory reaction of the host to bacteria present under the gums [1,2]. The gingival fibroblast plays an important role as a physical barrier against bacterial invasion and forms a part of the innate immune response against infection. Gingival fibroblasts produce several cytokines, chemokines, and matrix metalloproteases in response to external stimuli, including bacterial pathogen-associated molecular patterns such as lipopolysaccharides (LPS) and peptidoglycan (PGN) and cytokines (IL-1β and TGF-β) [3]. Gingival fibroblasts can also regulate the lodging or retention of lymphocytes in periodontitis lesions by activating various adhesion molecules [2]; pro-inflammatory cytokines are closely involved in enabling this process [4]. Thus, the regulation of the functioning of gingival epithelial cells by targeting the immune response might prevent the onset of periodontal disease.

IL-1 is one of the most potent pro-inflammatory cytokines and is mainly produced by macrophages. It is a well-known inducer of IL-6 and IL-8 in many types of cells, such as fibroblasts, endothelial cells, and keratinocytes [5,6]. It is the major cytokine produced at inflamed sites and is involved in the progression of connective tissue destruction [3]. It plays a critical role in the regulation of inflammation and immunologic reactions by activating a variety of genes and by affecting the function and growth of various cells [7]. IL-1 is a prominent cytokine that has been proposed to act as a potential predictor of periodontal disease progression in the periodontal tissue and gingival crevicular fluid [8,9]. Several genes downstream of IL-1β that have been identified in gingival fibroblasts are involved in periodontitis development [10]. Thus, many researchers consider IL-1 a therapeutic target for the treatment of periodontal diseases and have studied its synthesis, secretion, and biological signaling pathways [11].

IL-1β signaling is initiated by the binding of IL-1β to the type I receptor (IL-1R1), and this process is promoted by the co-receptor IL-1 receptor accessory protein (IL-1RAP), which causes the formation of a trimeric complex [12]. IL-1β binds to two types of receptors, i.e., IL-1R1 and IL-1R2. IL-1R1 is mostly expressed in fibroblasts, endothelial cells, and T cells, and IL-1R2 is predominantly expressed in B cells, macrophages, and neutrophils [13]. Inflammatory signaling via IL-1R can be mediated by the binding of IL-1R to either the activating cytokines or antagonistic ligands such as IL-1Ra, which inhibit downstream signaling [14]. Several promising molecules could reportedly modulate IL-1 signaling as a therapeutic strategy, including anakinra, a slightly modified IL-1R recombinant protein that competitively blocks IL-1 signaling [15], several IL-1R antagonist peptides [16,17], and IL-1-neutralizing antibodies [18]. Thus, many studies have focused on achieving IL-1 activity modulation by blocking the direct interactions between IL-1β and IL-1R.

In recent years, a number of pharmacophore-based virtual screening approaches have been applied for the identification of inhibitors or boosters of specific receptors (TLRs) or cytokines (IL-15 and IL-18) [19,20,21]. The crystal structures of IL-1β and of the IL-1β–IL-1R complex have been determined [22], which provides the advantage of enabling the prediction of potential molecules that might influence IL-1β signaling via computational analysis. Among the various cytokines produced by cells, IL-6 is known to be a multifunctional cytokine that can be produced by IL-1β–IL-1R signaling, and is very important for the prevention or treatment of skin aging [23]. IL-6 plays an active role in epidermal regeneration and also activates cellular and humoral immunity [24]. However, there are few reports about small molecules that enhance IL-1β–IL-1R-dependent IL-6 production in oral cells [2].

Here, we employed a structure-based virtual screening to identify small molecules that might bind to IL-1R1 and enhance human IL-1β–IL1R1 interaction. Sixty compounds that were identified by in silico screening at the site of modulation in ILR were tested to determine if they activated or repressed IL-1β signaling in GFs. We found that DPIE augmented pro-inflammatory cytokine production in IL-1β-stimulated GFs by enhancing the interactions between IL-1β and IL-1R1.

## 2. Results

### 2.1. Virtual Screening of IL-1R-Binding Small Molecules and Screening of the Strongest Hit

**Figure 1:**
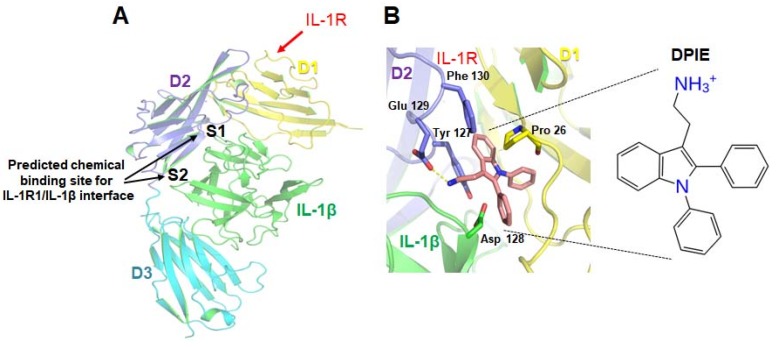
Prediction of the small molecules capable of regulating interleukin-1 β (IL-1β)–interleukin-1 receptor, type I (IL-1R1) interactions. (**A**) Tertiary structure model of the complex formed by IL-1β and the interleukin-1 receptor, type I. The three Ig-like domains (D1, D2, D3) of IL-1R1 and IL-1β are shown in yellow, purple, cyan, and green. A virtual screening study was performed for site 1 (S1) and site 2 (S2). (**B**) A potential docking model for 2-(1,2-diphenyl-1H-indol-3-yl)ethanamine (DPIE) at the IL-1β–IL-1R1 binding interface and DPIE structure. The dotted yellow line represents a hydrogen bond formed between DPIE and Glu129 in the IL-1 receptor, type 1(IL-1R1).

Using in silico computational analysis as described in Materials and Methods (Figure 1), we obtained a list of 60 chemicals that might potentially bind to IL-1R1. We examined all of them to see whether any of these chemicals affected pro-inflammatory cytokine production in IL-1β-stimulated GFs. Of 60 chemicals, only 2-(1,2-diphenyl-1H-indol-3-yl)ethanamine (DPIE) caused a dramatic change in pro-inflammatory cytokine expression in IL-1β-stimulated GFs (Supplementary Table S1). Treatment of IL-1β-stimulated GFs with 4 μM DPIE significantly increased IL-6 and IL-8 production by 3-fold and 2-fold, respectively, compared to the control (GFs stimulated with IL-1β only).

### 2.2. The Effect of DPIE on Pro-Inflammatory Cytokine Production in IL-1β-Stimulated GFs

**Figure 2:**
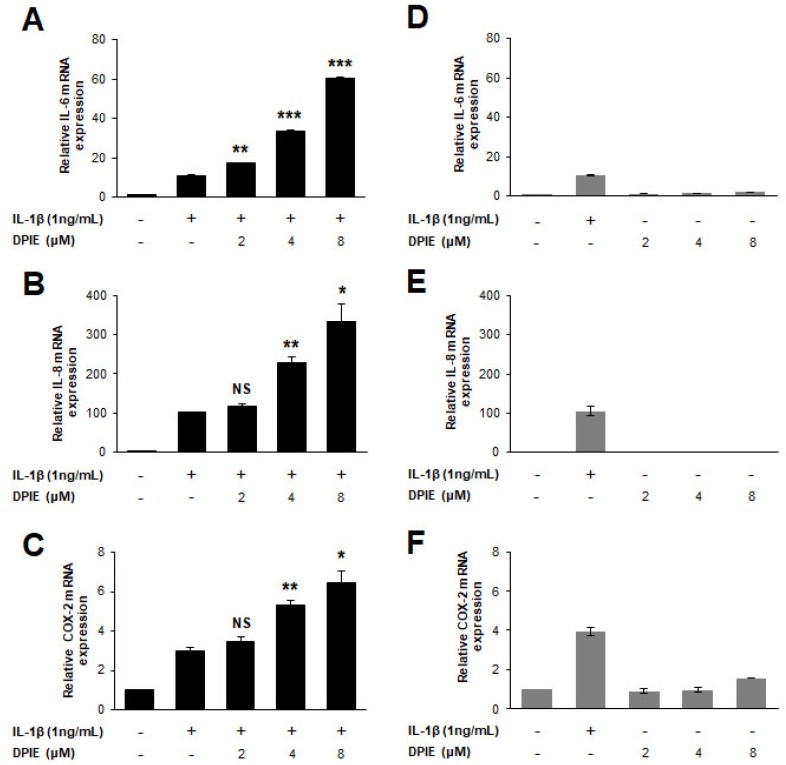
DPIE enhances pro-inflammatory cytokine expression in IL-1β-stimulated hGFs. hGFs were treated for 12 h with 0, 2, 4, or 8 μM DPIE in the absence or presence of 10 ng/mL IL-1β. (**A**–**C**) Concentration-dependent enhancing effects of DPIE on IL-6, IL-8, and COX-2 mRNA in IL-1β-stimulated hGFs. (**D**–**F**) The effect of DPIE alone on IL-6, IL-8, and COX-2 mRNA in hGFs. * *p* < 0.05, ** *p* < 0.01, *** *p* < 0.001, and NS: not significant compared with IL-1β alone (unpaired two-tailed Student’s *t* tests).

After screening for chemicals showing an impact on cytokine expression in IL-1β-stimulated GFs, we selected DPIE for further testing. To determine whether DPIE enhances inflammation in IL-1β-stimulated GFs in a concentration-dependent manner, we first tested the mRNA expression level of pro-inflammatory cytokines (IL-6 and IL-8) and COX-2 in IL-1β-stimulated GFs treated with DPIE at different concentrations, i.e., 0, 2, 4, and 8 μM. We found that the mRNA expression of IL-6, IL-8, and COX-2 was induced by DPIE in a concentration-dependent manner (Figure 2A–C). Furthermore, to demonstrate that the effect of DPIE depends on IL-1β signaling, we tested inflammation in GFs with DPIE alone at the different indicated concentrations. As shown in Figure 2D–F, DPIE alone did not affect inflammation in GFs. In addition, the expression of neither iNOS nor Tumor Necrosis Factor-α (TNF-α) was affected by IL-1β or DPIE (Supplementary Figure S1).

**Figure 3:**
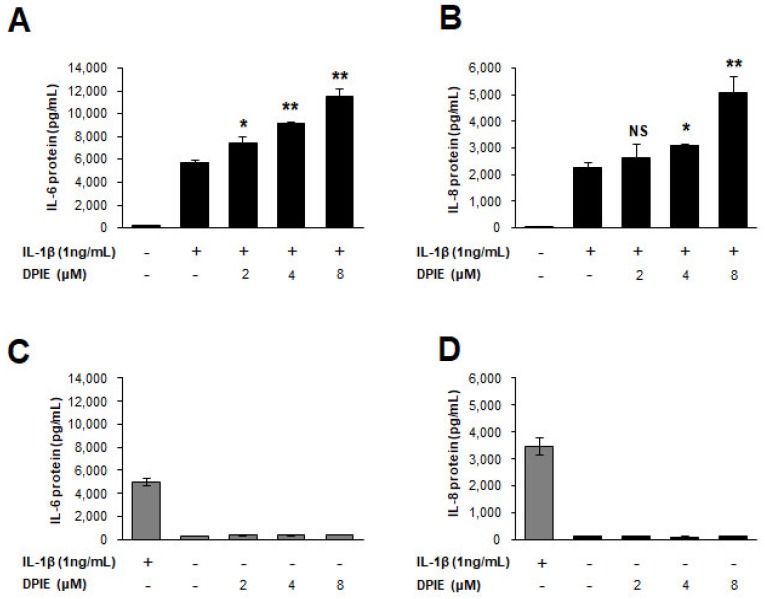
DPIE enhances pro-inflammatory cytokine secretion in IL-1β-stimulated human gingival fibroblasts (hGFs). hGFs were treated for 12 h with 0, 2, 4, or 8 μM DPIE in the absence or presence of 10 ng/mL IL-1β, and the supernatants were analyzed using ELISA to detect the presence of IL-6 and IL-8. Concentration-dependent enhancing effects of DPIE on IL-6 protein (**A**) and IL-8 protein (**B**) produced in IL-1β-stimulated hGFs. The effect of DPIE alone on IL-6 protein (**C**) and IL-8 protein (**D**) production in hGFs; * *p* < 0.05, ** *p* < 0.01; NS: not significant compared with IL-1β alone (unpaired two-tailed Student’s *t* tests).

Next, to test the protein expression level of pro-inflammatory cytokines (IL-6 and IL-8) affected by DPIE in IL-1β-stimulated GFs, we performed ELISA to detect human IL-6 and IL-8 in cell culture supernatants. Figure 3 shows that IL-6 and IL-8 protein production increased in a DPIE-concentration dependent manner in IL-1β-stimulated GFs, but DPIE alone did not affect IL-6 and IL-8 protein production in GFs. Notably, there were no cytotoxic effects observed because of DPIE treatment in IL-1β-stimulated or unstimulated GFs (Supplementary Figure S2).

Moreover, to confirm that the effect of DPIE on cytokine production in GFs is only dependent upon IL-1β stimulation, we tested the effect of DPIE in GFs under bacterial infectious condition. We infected GFs with *Fusobacterium nucleatum*, which is known to be a cytokine inducer [25] in the absence or presence of DPIE at different concentrations. Supplementary Figure S3 shows that DPIE did not affect *F. nucleatum*-induced pro-inflammatory cytokine production in GFs.

### 2.3. The Effect of DPIE on Pro-Inflammatory Cytokine Production in IL-1β-Stimulated PDLs

**Figure 4:**
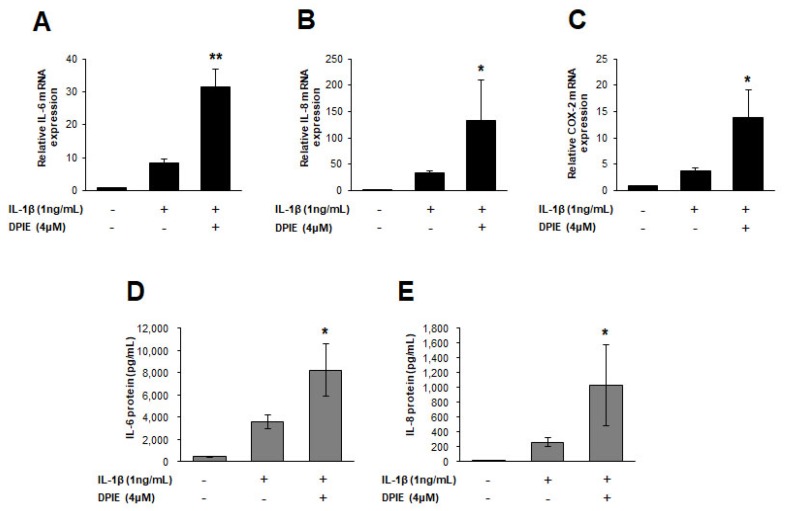
DPIE enhances pro-inflammatory cytokine expression in IL-1β-stimulated human periodontal ligament cell (hPDLs). hPDLs were treated for 12 h with 4 μM DPIE in the absence or presence of 10 ng/mL IL-1β, and then mRNAs and supernatants were analyzed using real-time PCR and ELISA, respectively. (**A**–**C**) DPIE enhances IL-6, IL-8, and COX-2 mRNA production in IL-1β-stimulated hPDLs. (**D**,**E**) DPIE enhances IL-6 and IL-8 protein production in IL-1β-stimulated hPDLs; * *p* < 0.05 and ** *p* < 0.01 compared with IL-1β alone (unpaired two-tailed Student’s *t* tests).

To investigate whether the cytokine enhancing effect of DPIE is replicated in IL-1β-stimulated PDLs, we treated IL-1β stimulated PDLs with 4 μM of DPIE, and DPIE-untreated PDLs were used as a control. Treatment of IL-1β-stimulated PDLs with 4 μM DPIE caused IL-6, IL-8, and COX-2 production to become three times higher than that observed in the DPIE-untreated control (Figure 4A). The relative increase of IL-6 and IL-8 levels in PDLs after treatment with 4 μM DPIE was the same as that observed in GFs. We also confirmed that IL-6 and IL-8 protein levels were increased in the culture supernatants of IL-1β-stimulated PDLs treated with 4 μM DPIE (Figure 4B).

### 2.4. Surface Plasmon Resonance (SPR) Data

**Figure 5:**
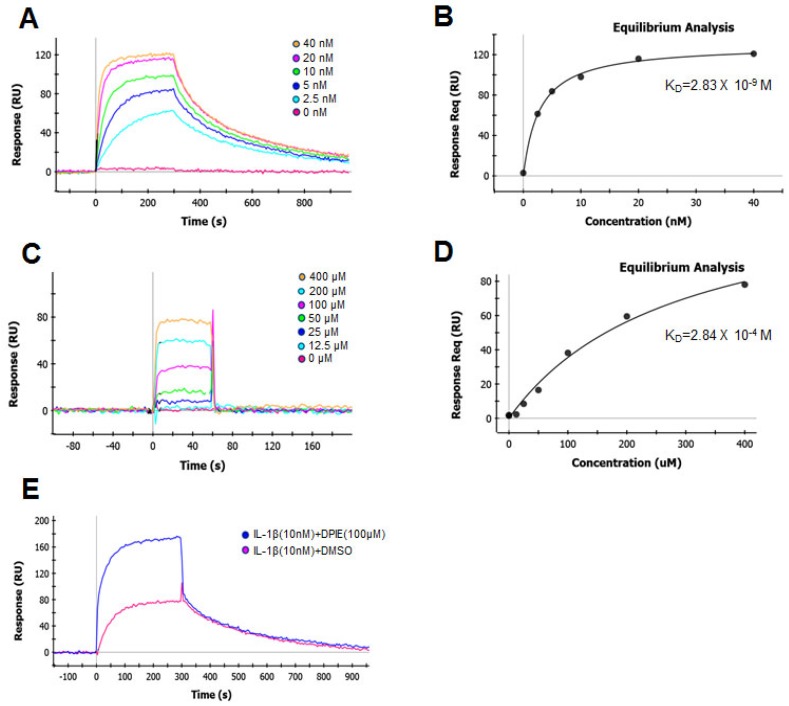
Global kinetic and equilibrium analysis of IL-1β or DPIE bound to IL-1R1 immobilized on a biosensor surface. Binding of increasing concentrations of IL-1β (2.5, 5, 10, 20, and 40 nM) to IL-1R (**A**) and its equilibrium analysis curve (**B**). Binding of increasing concentrations of DPIE (12.5, 25, 50, 100, 200, and 400 μM) to IL-1R (**C**) and its equilibrium analysis curve (**D**). Binding of IL-1β (10 nM) alone or IL-1β and DPIE (100 μM) to IL-1R1 (**E**). The dissociation constant (K_D_) value derived from the global fit of a single binding experiment is indicated with the fitting error, shown in parentheses.

To understand the molecular mechanism by which DPIE enhanced IL-1β signaling, we used SPR to determine the impact of this chemical on the binding of IL-1β to the main purified component (IL-1R1) of the IL-1β signaling system. We first determined the kinetics of the interaction and the binding affinity between IL-1β and IL-1R1 immobilized on biosensor chips (Figure 5A). The data were analyzed according to a 1:1 Langmuir binding model [26]. We analyzed the sensograms after injecting six concentrations of IL-1β into the captured IL-1R1. This analysis yielded an association rate constant (k_a_) of 2.16 × 10^6^ M^−1^ S^−1^ and a dissociation rate constant (k_d_) of 3.79 × 10^−3^ S^−1^ (Table 1). The equilibrium dissociation constant K_D_, derived from multiple measurements using various concentration of IL-1β, was 1.76 nM (Table 1). These binding affinity values are similar to previously reported measured values for this interaction that were determined using a variety of cell-based assays [27].

Next, we assessed the binding affinity of the interaction between DPIE and IL-1R1 immobilized on biosensor chips (Figure 5B). The association rate constant (k_a_) and dissociation rate constant (k_d_) of DPIE were 1.87 × 10^3^ M^−1^ S^−1^ and 5.91 × 10^−1^ S^−1^, respectively. The equilibrium dissociation constant K_D_ for the interaction between DPIE and IL-1R1 was 0.316 mM. The association rate constant for the binding of DPIE to IL-1R1 was ~1000-fold slower than that observed for the binding of IL-1β to IL-1R (Table 1), and the rate of dissociation of DPIE from IL-1R1 was ~100-fold faster than that of IL-1β to IL-1R1. The equilibrium dissociation constant K_D_ of the interaction between DPIE and IL-1R1 was ~170,000-fold higher than that of the interaction between IL-1β and IL-1R1. Moreover, by performing equilibrium analysis on the SPR data for the interaction of IL-1β or DPIE with IL-1R1, we obtained K_D_ values of 2.83 nM and 0.284 mM, respectively (Figure 5B,D). Taken together, it can be inferred that DPIE is capable of binding to IL-1R1, but its binding is very weak compared to that of IL-1β to IL-1R1.

To confirm that DPIE enhances IL-1β–IL-1R1 binding process, we performed the SPR assay for two conditions, i.e., for the interaction of IL-1β with IL-1R1 and of IL-1β and DPIE with IL-1R1. As shown in Figure 5E, the binding affinity of IL-1β and DPIE to IL-1R1 was dramatically increased, compared to that of IL-1β to IL-1R1. Therefore, we suggest that the addition of DPIE during the interaction between IL-1β and IL-1R1 enhances IL-1β–IL-1R1 binding and potentially influences IL-1β signaling.

## 3. Discussion

Chemical mediators of inflammation such as cytokines and chemokines are important for the defense of the host against bacterial infection [28]. Periodontal diseases are caused by gram-negative bacterial infections and pro-inflammatory cytokines, including IL-6, IL-8, and IL-1β, which appear to be major mediators of inflammation in periodontitis [29]. IL-6 is a multifunctional cytokine that plays a central role in host defense because of its wide range of immune and hematopoietic activities [30]. The IL-8 chemokine plays an important role in the initiation and development of inflammatory processes via its capacity to attract and activate neutrophils [31]. IL-8 level was positively correlated with IL-6 level in patients with periodontitis [29]. These pro-inflammatory cytokines, IL-6 and IL-8, can be produced by IL-1β stimulation in human GFs and PDLs.

IL-1β is an important target in the regulation of inflammation-related diseases; there have been numerous studies regarding small molecules that affect IL-1 signaling by hindering the interactions between IL-1β and IL-1R [2,17,32,33,34]. Because many studies have been conducted using an IL-1R antagonist, which directly binds to IL-1R and influences IL-1β–IL-1R interactions, in the present study, small molecules that potentially bind to IL-1R were designed using a computational approach. We found that DPIE alone showed a weak binding activity towards IL-1R that was not enough to trigger IL-1β signaling (Figure 5). However, interestingly, the binding of DPIE to IL-1R caused IL-1β to strongly bind to IL-1R (Figure 5), which subsequently led to the activation of IL-1β signaling and its downstream (cytokine production) outcomes; no cytotoxic effects were observed during these process (Figure 2 and Figure 3, Supplementary Figure S2). Moreover, we observed that DPIE had no impact on *Fusobacterium nucleatum*-induced cytokine production in GFs (Supplementary Figure S3). Thus, we inferred that DPIE boosted cytokine production in GFs in an IL-1β signaling-dependent manner but not in a TLR4-mediated signaling-dependent manner, as we had hypithesized at the beginning.

The majority of studies regarding the regulation of molecules for IL-1β signaling have shown that the inhibition of IL-1β signaling is beneficial to hosts with inflammatory diseases such as rheumatoid arthritis, osteoarthritis, and chronic systemic inflammatory conditions [35,36,37]. In contrast, few studies have demonstrated the positive impact of small molecules that synergistically activate IL-1β signaling on the defense mechanism of the host [2,38,39]. Evidence supporting the latter points corroborate our finding regarding the role of DPIE in IL-1β signaling. For example, Yu et al. reported that the host defense peptide LL-37 synergistically enhanced IL-1β-induced production of cytokines (IL-6, IL-10) and chemokines (MCP-1, MCP-3) in human peripheral blood mononuclear cell (PBMCs), which suggests that they have a role in enhancing certain innate immune responses [38]. In addition, the activity of IL-1β was positively correlated with antimicrobial activity in macrophages [39]. Because the increased activity of IL-1β is consistent with its increased affinity for IL-1R, the enhancing role of DPIE in the activation of IL-1β signaling might potentially increase the antimicrobial activity of phagocytic immune cells. Thus, in future studies, it would be interesting to investigate the function of DPIE in immune cells acting against intracellular pathogens.

Human gingival fibroblasts can play an important role in regulating local inflammation by producing cytokines and chemical mediators. According to Murakami et al. (2001), adenosine and its agonists synergistically increased IL-1β-induced IL-6 and IL-8 production by interacting with adenosine receptors that affect the cAMP–protein kinase A pathway [2]. Although the mechanism leading to the increase of cytokines is significantly different from that observed in our study, the finding about the cytokine boosting effect in GFs is in accordance with our findings.

It is well known that IL-1β is the key inflammatory cytokine in periodontal diseases and that the elevated expression of IL-1β is closely related to the manifestations of the disease [40]; hence, GFs in inflamed periodontal lesions are considered to be seriously affected by locally secreted IL-1β [2]. Although the present study reports that the DPIE enhanced cytokine production in IL-1β-induced GFs, further investigations are still necessary to clarify whether DPIE has beneficial or harmful effects on the progression of periodontal diseases in vivo. Nevertheless, this study proves that the chemical DPIE can enhance the binding of IL-1β to IL-1R1 and consequently enhance the activation of IL-1β signaling and subsequent cytokine release.

## 4. Materials and Methods

### 4.1. Ethics Statement

The isolation of human GFs and PDLs was approved by the Chonnam National University Dental Hospital Institutional Review Board (approval No. CNUDH-2016-013, 20 October 2016). Written informed consent was obtained from all subjects after the nature and possible consequences of the studies were explained to them. All participants were adults who did not have periodontal disease.

### 4.2. Cell Culture

Human GF and PDL cells were cultured in Dulbecco’s modified Eagle’s medium (DMEM, Gibco BRL, Grand Island, NY, USA) supplemented with 10% heat-inactivated fetal bovine serum (PAA Laboratories, Etobicoke, ON, Canada), 100 U/mL penicillin, and 100 μg/mL streptomycin (Gibco BRL) at 37 °C in a humidified atmosphere containing 5% CO_2_. When confluent, the cells were trypsinized using a 0.25% trypsin/0.02% EDTA solution (Sigma, St. Louis, MO, USA).

### 4.3. Reagents

All 60 chemicals were used without carrying out further purification and were purchased from two commercial sources (Hit2lead, San Diego, CA, USA; Chemdiv, San Diego, CA, USA). Detailed information about the chemicals used in this study is available in Supplementary Table S1.

### 4.4. Virtual Screening of Small Molecular Regulators of IL-1β–IL1R1 Interactions

To identify small molecules capable of regulating IL-1β–IL-1R1 interactions, we performed a structure-based virtual screening of a commercially available library. The crystal structure of the IL-1β–IL-1R1 complex (PDB ID 4GAF) was obtained from the protein databank and was prepared for analysis using the protein preparation wizard of the Schrodinger program (Schrodinger LLC, 2017-4, New York, NY, USA). Water molecules were deleted, and hydrogen atoms were added. A restrained minimization was then performed with the OLLS3 force field using the default constraint of 0.30 Å RMSD. Site analysis was performed to assess IL-1β–IL-1R1 interactions using the Schrodinger Package’s SiteMap tool, to analyze compounds that could bind to the interface. As a result, it was predicted that the pro-26 residue of IL-1R1 and the Tyr 127 residue could potentially bind to regulator molecules. Then, the IL-1β–IL-1R1 interaction interface was defined, and a grid file was generated using the receptor grid generation panel. The virtual screening of the chemical library was performed with prepared proteins using the OPLS3 force field, generated using the Virtual Screening Workflow tool from the Schrodinger Package. VSW uses Glide docking to rank the compound that utilizes the scoring functions in the most effective manner. As shown in Figure 1A, we tried to perform a virtual screening study by analyzing Site 1 and Site 2. The library of compounds used in the virtual screening study contained 1.5 million compounds sourced from ChemBridge’s library (available online: http://www.hit2lead.com); these compounds are commercially available.

### 4.5. Real-Time PCR

Total RNA was isolated using RNeasy kits (Qiagen, Hilden, Germany) primed with random hexamer oligonucleotides and was reverse transcribed using a PrimeScriptTM RT reagent kit (Takara, Japan). Real-time PCR was performed using an ABI 7300 Prism SDS real-time PCR detection system (Applied Biosystems, Foster City, CA, USA) with a SYBR^®^ Premix Ex Tag kit (Takara Biotechnology, Shiga, Japan) and a standard temperature protocol. The results obtained using a cycle threshold are expressed as relative quantities and were calculated using the 2^−ΔΔ*C*T^ method (expressed as the relative fold ratio). All data were normalized to human GAPDH data, and three separate experiments were performed. Supplementary Table S2 lists the primers used during quantitative real-time PCR.

### 4.6. ELISA

Human GFs and PDLs were harvested at the fourth passage and seeded at a density of 0.8 × 10^5^ cells in 12-well culture plates. When the cells attained 90 to 100% confluence, they were incubated with DPIE at the indicated concentration in the presence or absence of IL-1β (1 ng/mL) for 12 h. At the end of the incubation period, the supernatants were collected and stored at −20 °C until IL-6 and IL-8 levels were determined. The IL-6 and IL-8 levels in the culture supernatants were measured by using an ELISA kit (Bio legend, San Diego, CA, USA), in accordance with the manufacturer’s protocol.

### 4.7. Cell Viability Assay

The viability of the cultured cells was measured using the MTT colorimetric assay (Roche Diagnostics, Meylan, France), as described previously [7]. In brief, the culture medium was replaced with MTT (0.2 mg/mL) dissolved in DMEM, and the cells were incubated for 2 h at 37 °C. Then, 100 μL of solubilization solution (10% SDS (g/mL) in 0.01 M HCL) was added, and the plates were allowed to stand overnight at 37 °C in a humidified atmosphere.

Cell viability was directly related to the difference in the absorbance measured at 540 nm using a SpectaMax i3X (San Jose, CA, USA) micro-titer plate reader. The results were normalized relative to their respective controls.

### 4.8. Surface Plasmon Resonance (SPR)

All SPR binding experiments were performed on the ProteOn XPR36 system (Bio-Rad Laboratories, Hercules, CA, USA) instrument at 25 °C. Phosphate buffer saline (PBS) containing 0.05% Tween-20 (pH 7.4) was used as the running buffer. IL-1R used as the ligand was immobilized on the GLH sensor chip using the standard amine-coupling method. IL-1R (5 µg/mL, 10 mM sodium acetate, pH 4.0) was injected for 5 min at a flow rate of 30 µL/min over the surface, after pre-activation with a mixture of 0.2 M of 1-ethyl-3-(3-dimethylaminopropyl) carbodiimide hydrochloride (EDCA) and 0.05 M of N-hydroxysulfosuccinimide (sulfo-NHS). After injecting the IL-1R protein, the surface was deactivated with 1 M of ethanolamine HCl, pH 8.5. Interactions between the small molecule test compounds and the immobilized protein were analyzed using PBS containing 0.05% Tween 20, 1% (*v*/*v*) DMSO, and the flow rate was set to 100 µL/min. Interactions between IL-1 beta and IL-1R were analyzed using PBS containing 0.05% Tween 20, and the flow rate was set to 30 µL/min. The data were analyzed with the ProteOn Manager software 3.1 (Bio-Rad Laboratories). The values of the dissociation (k_d_) and association (k_a_) rate constants, and the dissociation equilibrium constant (K_D_) were determined using the Langmuir 1:1 bimolecular kinetic model.

### 4.9. Statistical Analyses

Statistical analyses were performed using unpaired two-tailed Student’s *t* tests (* *p* < 0.05; ** *p* < 0.01; ** *p* < 0.001; NS, not significant). All data are expressed as the mean ± SD values. The results are representative of data from more than three independent experiments, with each experiment performed in triplicate.

## 5. Conclusions

In this study, we performed a virtual in silico screening to identify small molecules that could regulate IL-1β–IL-1R1 interactions. Out of 60 chemicals, only DPIE upregulated IL-1β-induced cytokine production in GFs and PDLs, because of its ability to augment IL-1β signaling by influencing IL-1β–IL-1R1 binding.

## Figures and Tables

**Table 1:** Kinetic parameters of the binding of IL-1β and DPIE to IL-1R1, analyzed by surface plasmon resonance (SPR).

Immobilized Ligand	Binding Interaction	k_a_	k_d_	K_D_
**IL-1R1**	IL-1β	2.16 × 10^6^	3.79 × 10^−3^	1.76 × 10^−9^
	DPIE	1.87 × 10^3^	5.91 × 10^−1^	3.16 × 10^−4^

## Supplementary Materials

Supplementary materials can be found at http://www.mdpi.com/1422-0067/19/7/1835/s1.

## Abbreviations

TLRs	Toll-like receptors
PGN	peptidoglycan
TGF-β	transforming growth factor beta
hGFs	human gingival fibroblasts
hPDLs	human periodontal ligament fibroblasts
DPIE	2-(1,2-diphenyl-1H-indol-3-yl)ethanamine
IL-1	interleukin-1
IL-1R1	interleukin-1 receptor, type I
TNF-α	Tumor Necrosis Factor-α
SPR	surface plasmon resonance
ELISA	Enzyme-linked immunosorbent assay
iNOS	inducible nitric oxide synthase
PBMC	peripheral blood mononuclear cell
MCP-1	monocyte chemoattractant protein-1
MCP-3	monocyte chemoattractant protein-3
MTT	3-(4,5-Dimethylthiazol-2-Yl)-2,5-Diphenyltetrazolium Bromide
