# Supplementary material for: DPIE [2-(1,2-diphenyl-1H-indol-3-yl)ethanamine] Augments Pro-Inflammatory Cytokine Production in IL-1β-Stimulated Primary Human Oral Cells
Source: Int J Mol Sci. 2018 Jun 22;19(7):1835. doi: 10.3390/ijms19071835 (PMC6073580; doi:10.3390/ijms19071835)
Supplement: Supplementary file 1 [file ijms-19-01835-s001.zip › ijms-321468 supplementary materials/figure caption.pdf]

Supplementary Figure A1.

The effects of DPIE on iNOS and TNF-α expression in IL-1β-stimulated hGFs. hGFs were treated for 12 h with 0, 2, 4, or 8 μM DPIE in the absence or presence of 10 ng/ml IL-1β. (A, B) The effects of DPIE on iNOS and TNF-α mRNA production in IL-1β-stimulated hGFs. (C, D) The effect of DPIE alone on iNOS and TNF-α mRNA production in hGFs.

Supplementary Figure A2.

Effects of DPIE on the viability of unstimulated and interleukin (IL)-1β-stimulated hGFs. hGFs were incubated for 12 h without (control) or with 10 ng/ml IL-1β in the presence of the indicated concentrations of DPIE, and the relative viable cell number was determined using the 3-[4,5-dimethylthiazol-2-yl]-2, 5-diphenyltetrazolium bromide method. Each value represents the mean ± SD of triplicate assays. The Student's *t*-test was used for comparing values between groups.

Supplementary Figure A3.

DPIE did not affect the proinflammatory cytokine expression in *F. nucleatum*-infected hGFs. hGFs were treated for 12 h with 0, 2, 4, or 8 μM DPIE in the absence or presence of *F. nucleatum* (MOI100). (A-F) DPIE did not affect IL-6, IL-8, COX2, iNOS, and TNF-α mRNA production in *F. nucleatum*-infected hGFs.
