# Supplementary material for: DPIE [2-(1,2-diphenyl-1H-indol-3-yl)ethanamine] Augments Pro-Inflammatory Cytokine Production in IL-1β-Stimulated Primary Human Oral Cells
Source: Int J Mol Sci. 2018 Jun 22;19(7):1835. doi: 10.3390/ijms19071835 (PMC6073580; doi:10.3390/ijms19071835)
Supplement: Supplementary file 1 [file ijms-19-01835-s001.zip › ijms-321468 supplementary materials/Supplementary Figures.pptx]

## Slide 1
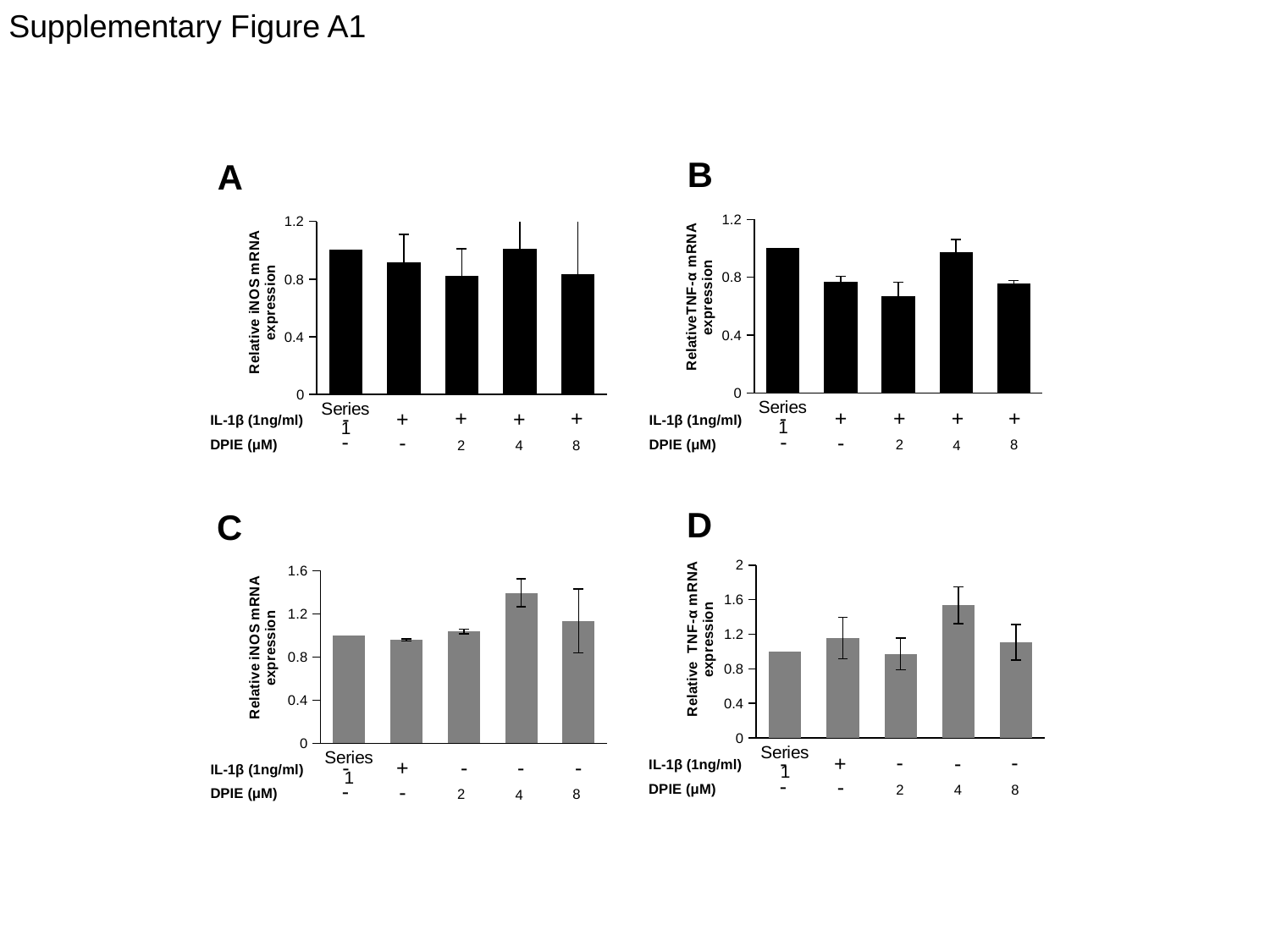

Supplementary Figure A1
B
A
### Chart
| Category | IL-1b (1ng/ml) + #5 |
|---|---|
| | 1.0 |
| | 0.763922334384483 |
| | 0.6652098212247235 |
| | 0.9730784303412079 |
| | 0.7526386849481073 |
### Chart
| Category | IL-1b (1ng/ml) + #5 |
|---|---|
| | 1.0 |
| | 0.9130094026391518 |
| | 0.8171457823456914 |
| | 1.0087696413415443 |
| | 0.8314955699192388 |+
+
+
+
+
+
-
+
+
-
IL-1β (1ng/ml)
IL-1β (1ng/ml)
-
-
-
-
DPIE (μM)
DPIE (μM)
2
8
4
2
8
4
D
C
### Chart
| Category | #5 alone |
|---|---|
| | 1.0 |
| | 1.156671277778803 |
| | 0.972625102492298 |
| | 1.5341006715962346 |
| | 1.1080942742475635 |
### Chart
| Category | #5 alone |
|---|---|
| | 1.0 |
| | 0.9579416619632931 |
| | 1.036514215633089 |
| | 1.3940410227586766 |
| | 1.1340459519316406 |-
-
+
-
-
-
-
+
-
-
IL-1β (1ng/ml)
IL-1β (1ng/ml)
-
-
-
-
DPIE (μM)
2
8
4
DPIE (μM)
2
8
4

## Slide 2
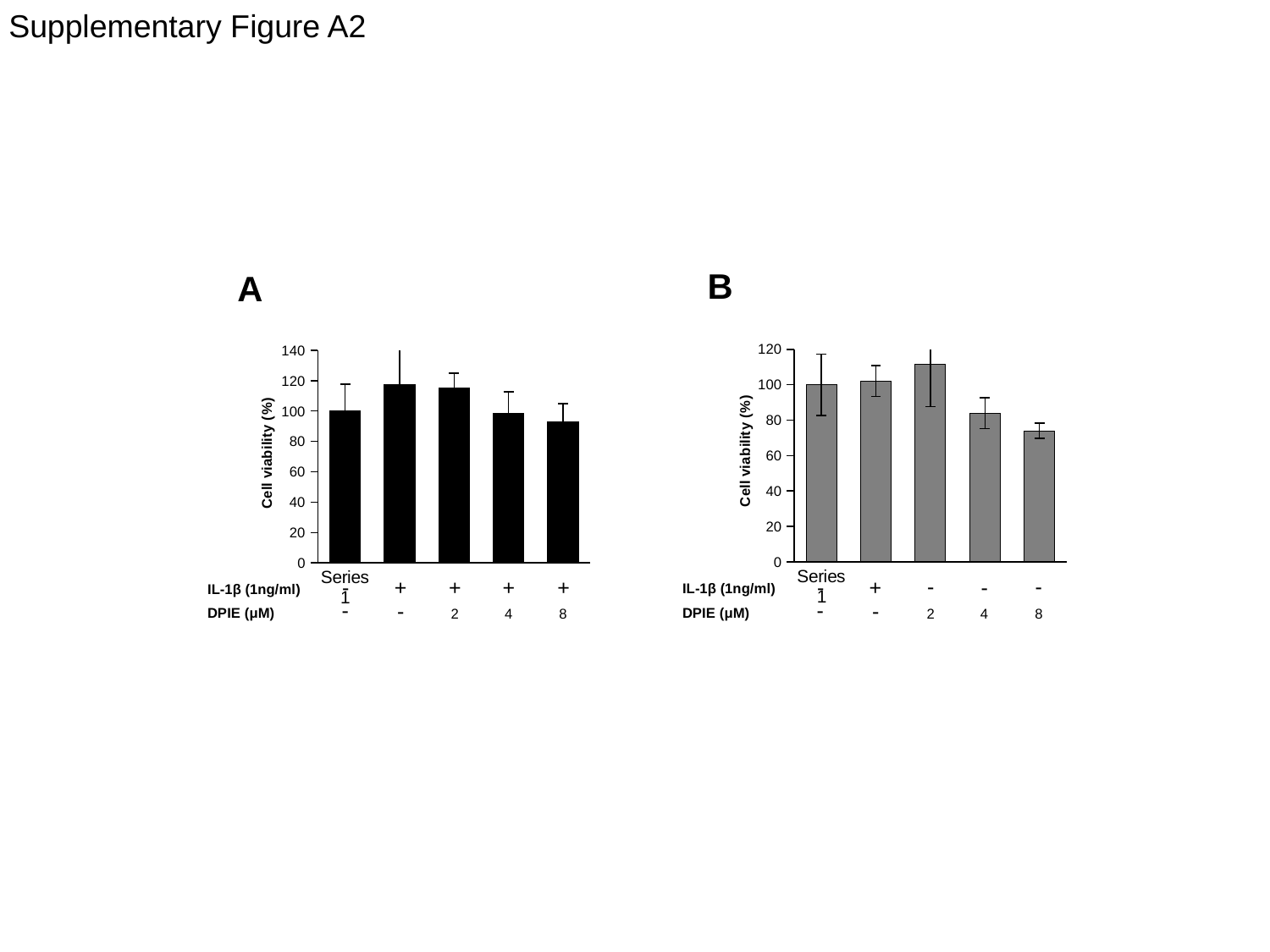

Supplementary Figure A2
B
A
### Chart
| Category | MTT assay |
|---|---|
| | 100.0 |
| | 102.08125445473986 |
| | 111.51342361606085 |
| | 83.87740555951534 |
| | 73.98907103825137 |
### Chart
| Category | MTT assay |
|---|---|
| | 100.0 |
| | 117.57524983543772 |
| | 115.35515528693674 |
| | 98.56980432050744 |
| | 93.14822571958591 |-
-
+
-
+
+
-
+
+
-
IL-1β (1ng/ml)
IL-1β (1ng/ml)
-
-
-
-
DPIE (μM)
DPIE (μM)
2
8
4
2
8
4

## Slide 3
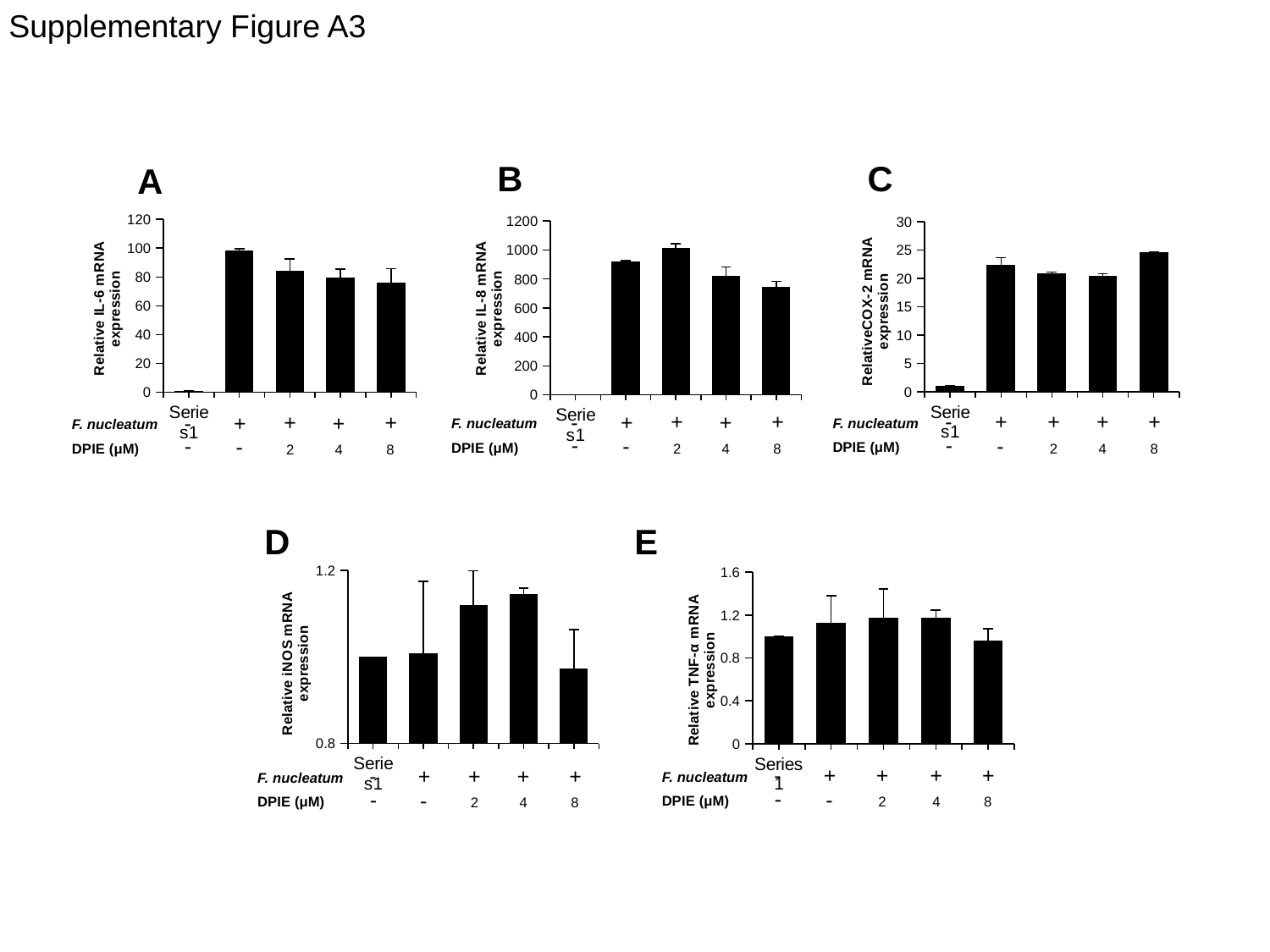

Supplementary Figure A3
B
C
A
### Chart
| Category | 1 22.45401942 20.97242692 20.42751206 24.58048663 |
|---|---|
### Chart
| Category | |
|---|---|
### Chart
| Category | |
|---|---|+
+
+
+
+
+
-
+
+
-
+
+
+
+
-
F. nucleatum
F. nucleatum
F. nucleatum
-
-
-
-
-
-
DPIE (μM)
DPIE (μM)
2
8
4
DPIE (μM)
2
8
4
2
8
4
D
E
### Chart
| Category | |
|---|---|
| | 1.0 |
| | 1.0092353745239535 |
| | 1.1208615664453105 |
| | 1.1461775132852958 |
| | 0.9737493810029954 |
### Chart
| Category | |
|---|---|
| | 1.0 |
| | 1.1235362665717912 |
| | 1.1738918183805123 |
| | 1.1728626956490582 |
| | 0.960666336640757 |+
+
+
+
-
+
+
+
+
-
F. nucleatum
F. nucleatum
-
-
-
-
DPIE (μM)
2
8
DPIE (μM)
4
2
8
4
