# Supplementary material for: DPIE [2-(1,2-diphenyl-1H-indol-3-yl)ethanamine] Augments Pro-Inflammatory Cytokine Production in IL-1β-Stimulated Primary Human Oral Cells
Source: Int J Mol Sci. 2018 Jun 22;19(7):1835. doi: 10.3390/ijms19071835 (PMC6073580; doi:10.3390/ijms19071835)
Supplement: Supplementary file 1 [file ijms-19-01835-s001.zip › ijms-321468 supplementary materials/Supplementary Table 1_sha.docx]

Supplementary Table 1. Chemicals used in this study.

| No. | **Chemical name** | **Relative ratio of IL6^a^**  **(Mean**$\boldsymbol{\pm SD}$**)** | **Relative ratio**  **of IL8^b^**  **(Mean**$\boldsymbol{\pm SD}$**)** | **Predicted binding site** |
| --- | --- | --- | --- | --- |
| 1 | 6-methoxy-2,2,4-trimethyl-1-[(4H-1,2,4-triazol-3-ylthio)acetyl]-1,2,3,4-tetrahydroquinoline | 0.51$\pm$0.35 | 0.84$\pm$0.84 | S1 |
| 2 | 1-(9H-xanthen-9-ylcarbonyl)-4-piperidinecarboxamide | 0.32$\pm$0.19 | 0.50$\pm$0.37 | S1 |
| 3 | N-[2-chloro-5-(trifluoromethyl)phenyl]-2-[(2-oxo-4-phenyl-2H-chromen-7-yl)oxy]acetamide | 0.91$\pm$0.52 | 1.70$\pm$1.24 | S2 |
| 4 | 1-(2-methyl-1H-indol-3-yl)-2-(4H-1,2,4-triazol-3-ylthio)ethanone | 0.36$\pm$0.18 | 0.35$\pm$0.18 | S1 |
| 5 | 2-(1,2-diphenyl-1H-indol-3-yl)ethanamine | 3.12$\pm$0.05*** | 2.22$\pm$0.14** | S1 |
| 6 | 2-[9-(3-methylbutyl)-9H-carbazol-3-yl]acetamide | 0.67$\pm$0.05 | 1.05$\pm$0.18 | S1 |
| 7 | 7-hydroxy-3'-methylspiro[chromene-2,1'-cyclohexan]-4(3H)-one | 0.90$\pm$0.07 | 1.14$\pm$0.01 | S1 |
| 8 | 3-(1,3-dioxooctahydro-2H-isoindol-2-yl)-4-(1-piperidinyl)benzamide | 0.80$\pm$0.15 | 0.94$\pm$0.29 | S1 |
| 9 | 2-{[1-(1-naphthylmethyl)-1H-benzimidazol-2-yl]thio}acetamide | 2.17$\pm$1.17 | 2.54$\pm$1.70 | S1 |
| 10 | 2-(4-chloro-3-methylphenoxy)-N-(3-pyridinylmethyl)propanamide | 0.46$\pm$0.43 | 0.52$\pm$0.47 | S2 |
| 11 | 2-(4-chloro-2-methyl-5-{[(3-pyridinylmethyl)amino]sulfonyl}phenoxy)acetamide | 0.78$\pm$0.59 | 0.91$\pm$0.69 | S2 |
| 12 | 4-[3-(4-chlorophenyl)-1,2,4-oxadiazol-5-yl]-N-(3-pyridinylmethyl)butanamide | 1.18$\pm$0.66 | 1.94$\pm$1.40 | S2 |
| 13 | 2-(4-bromo-2,5-dimethylphenoxy)-N-(3-pyridinylmethyl)acetamide | 0.65$\pm$0.61 | 0.60$\pm$0.52 | S2 |
| 14 | 2-(4-isopropylphenoxy)-N-(3-pyridinylmethyl)propanamide | 0.47$\pm$0.21 | 0.51$\pm$0.29 | S2 |
| 15 | 2-[2-chloro-4-(1-pyrrolidinylsulfonyl)phenoxy]-N-(3-pyridinylmethyl)acetamide | 2.17$\pm$1.15 | 1.49$\pm$0.35 | S2 |
| 16 | 3-{[2-(3-oxo-3,4-dihydro-2H-1,4-benzothiazin-2-yl)acetyl]amino}phenyl acetate | 0.74$\pm$0.34 | 0.61$\pm$0.27 | S2 |
| 17 | 3-(7-methoxy-4-methyl-2-oxo-2H-chromen-6-yl)-N-(3-pyridinylmethyl)propanamide | 0.57$\pm$0.26 | 0.56$\pm$0.33 | S2 |
| 18 | 2-(3-bromophenoxy)-N-(3-pyridinylmethyl)propanamide | 1.81$\pm$0.55 | 1.47$\pm$0.44 | S2 |
| 19 | 5-oxo-1-phenyl-N-(3-pyridinylmethyl)-3-pyrrolidinecarboxamide | 0.65$\pm$0.26 | 0.69$\pm$0.25 | S2 |
| 20 | 2-{4-[(dimethylamino)sulfonyl]-2-methylphenoxy}-N-(3-pyridinylmethyl)acetamide | 1.02$\pm$0.12 | 0.85$\pm$0.13 | S2 |
| 21 | 2-(4-chloro-3,5-dimethylphenoxy)-N-(3-pyridinylmethyl)propanamide | 0.77$\pm$0.07 | 1.06$\pm$0.34 | S2 |
| 22 | 2-{4-[methyl(methylsulfonyl)amino]phenoxy}-N-(3-pyridinylmethyl)propanamide | 0.81$\pm$0.19 | 1.00$\pm$0.26 | S2 |
| 23 | N-(4-chloro-3-fluorophenyl)-2-(3H-imidazo[4,5-b]pyridin-2-ylthio)acetamide | 0.48$\pm$0.10 | 0.63$\pm$0.22 | S2 |
| 24 | 1-[(4-bromophenyl)sulfonyl]-N-(3-pyridinylmethyl)-3-piperidinecarboxamide | 0.85$\pm$0.27 | 0.98$\pm$0.41 | S2 |
| 25 | 1-[(4-ethoxy-3-methylphenyl)sulfonyl]-N-(3-pyridinylmethyl)-3-piperidinecarboxamide | 0.82$\pm$0.09 | 0.91$\pm$0.06 | S2 |
| 26 | 1-{3-[(2,5-dimethylphenyl)amino]-3-oxopropyl}-4-piperidinecarboxamide | 1.08$\pm$0.32 | 1.19$\pm$0.31 | S2 |
| 27 | 4-(1-piperidinylsulfonyl)-N-(3-pyridinylmethyl)-2-thiophenecarboxamide | 1.17$\pm$0.30 | 1.41$\pm$0.25 | S2 |
| 28 | 4-[[(dimethylamino)sulfonyl](methyl)amino]-N-(3-pyridinylmethyl)benzamide | 1.47$\pm$0.04 | 1.44$\pm$0.12 | S2 |
| 29 | 1-[(dimethylamino)sulfonyl]-N-(3-pyridinylmethyl)-4-piperidinecarboxamide | 1.34$\pm$0.34 | 1.78$\pm$0.34 | S2 |
| 30 | 3-{4-[(dimethylamino)sulfonyl]phenyl}-N-(3-pyridinylmethyl)propanamide | 1.49$\pm$0.37 | 1.78$\pm$0.60 | S2 |
| 31 | 4-[(3-methylphenyl)(methylsulfonyl)amino]-N-(3-pyridinylmethyl)butanamide | 0.57$\pm$0.22 | 0.69$\pm$0.20 | S2 |
| 32 | N-ethyl-4-(2-oxo-1-pyrrolidinyl)-N-[2-(4-pyridinyl)ethyl]benzenesulfonamide | 0.57$\pm$0.21 | 0.66$\pm$0.29 | S2 |
| 33 | 4-[[(4-methoxyphenyl)sulfonyl](methyl)amino]-N-(4-pyridinylmethyl)benzamide | 0.75$\pm$0.33 | 0.99$\pm$0.59 | S2 |
| 34 | 5-(4-methoxy-1-phthalazinyl)-2-methyl-N-(3-pyridinylmethyl)benzenesulfonamide | 0.44$\pm$0.21 | 0.37$\pm$0.16 | S2 |
| 35 | 2-{4-[(isopropylamino)sulfonyl]-2-methylphenoxy}-N-(3-pyridinylmethyl)acetamide | 0.73$\pm$0.39 | 0.76$\pm$0.41 | S2 |
| 36 | 5-oxo-5-(3-oxo-1-piperazinyl)-N-(4-phenoxyphenyl)pentanamide | 0.56$\pm$0.13 | 0.60$\pm$0.13 | S2 |
| 37 | N-(1,3-benzodioxol-5-ylmethyl)-2-(3,5-dimethylphenoxy)propanamide | 1.08$\pm$0.28 | 1.36$\pm$0.64 | S2 |
| 38 | 3-methyl-4-oxo-N-(3-pyridinylmethyl)-3,4-dihydro-1-phthalazinecarboxamide | 1.24$\pm$1.04 | 1.40$\pm$0.90 | S2 |
| 39 | 2-(2-bromo-4-isopropylphenoxy)-N-(3-pyridinylmethyl)acetamide | 1.68$\pm$0.03 | 1.74$\pm$0.35 | S2 |
| 40 | N-[2-(3,4-dimethoxyphenyl)ethyl]-2-(3-oxo-2-piperazinyl)acetamide | 0.92$\pm$0.43 | 1.08$\pm$0.51 | S2 |
| 41 | tetrahydro-2-furanylmethyl 6-{[(4-acetylphenyl)amino]carbonyl}-3-cyclohexene-1-carboxylate | 0.81$\pm$0.19 | 0.84$\pm$0.10 | S2 |
| 42 | 4-oxo-4-(3-oxo-1-piperazinyl)-N-(4,5,6,7-tetrahydro-1,3-benzothiazol-2-yl)butanamide | 0.86$\pm$0.12 | 1.08$\pm$0.06 | S2 |
| 43 | 2-chloro-4-[methyl(methylsulfonyl)amino]-N-(3-pyridinylmethyl)benzamide | 0.77$\pm$0.12 | 0.54$\pm$0.05 | S2 |
| 44 | (2,4-dihydroxyphenyl)(4-hydroxyphenyl)methanone | 1.19$\pm$0.74 | 0.88$\pm$0.45 | S2 |
| 45 | 4-[3-(1,3-benzodioxol-5-ylamino)-6-methylimidazo[1,2-a]pyridin-2-yl]phenol | 0.90$\pm$0.18 | 0.76$\pm$0.19 | S2 |
| 46 | N-(3-acetylphenyl)-N'-{4-[2-(1-ethyl-1H-benzimidazol-2-yl)ethyl]phenyl}urea | 0.60$\pm$0.13 | 0.41$\pm$0.10 | S2 |
| 47 | N-(2,5-diethoxyphenyl)-1-[1-(3-fluorobenzyl)-1H-benzimidazol-2-yl]piperidine-4-carboxamide | 1.37$\pm$0.61 | 2.16$\pm$0.62 | S2 |
| 48 | 2,7-diamino-4-(4-fluorophenyl)-4H-chromene-3-carbonitrile | 0.47$\pm$0.08 | 0.83$\pm$0.16 | S2 |
| 49 | 4-[3-(1,3-benzodioxol-5-ylamino)-7-methylimidazo[1,2-a]pyridin-2-yl]phenol | 0.60$\pm$0.25 | 0.54$\pm$0.29 | S2 |
| 50 | N-(1-benzyl-2-oxo-1,2,3,4-tetrahydroquinolin-6-yl)acetamide | 0.63$\pm$0.23 | 0.43$\pm$0.09 | S2 |
| 51 | 2-[1-(4-hydroxyphenyl)cyclohexyl]phenol | 0.83$\pm$0.26 | 0.71$\pm$0.33 | S2 |
| 52 | 1-{[1-(4-fluorobenzyl)-1H-indol-2-yl]methyl}-N-(1-methyl-3-phenylpropyl)piperidine-4-carboxamide | 0.69$\pm$0.29 | 0.61$\pm$0.22 | S2 |
| 53 | N-(2-fluorophenyl)-N'-{3-[2-(1-phenyl-1H-benzimidazol-2-yl)ethyl]phenyl}urea | 0.87$\pm$0.19 | 0.74$\pm$0.22 | S2 |
| 54 | 4-[1-(4-hydroxybenzyl)-1H-benzimidazol-2-yl]phenol | 0.92$\pm$0.22 | 0.76$\pm$0.19 | S2 |
| 55 | 3-fluoro-N-{4-[2-(1-phenyl-1H-benzimidazol-2-yl)ethyl]phenyl}benzamide | 0.67$\pm$0.23 | 0.37$\pm$0.16 | S2 |
| 56 | **N-(3-fluorophenyl)-3-[(3-oxo-3,4-dihydro-2H-1,4-benzothiazin-6-yl)sulfonyl]propanamide** | 1.11$\pm$0.19 | 0.90$\pm$0.07 | S2 |
| 57 | N-(5-chloro-2-methylphenyl)-1-[1-(3-fluorobenzyl)-1H-benzimidazol-2-yl]piperidine-4-carboxamide | 1.38$\pm$0.04 | 1.03$\pm$0.12 | S2 |
| 58 | (2E,6Z)-2-(3-hydroxybenzylidene)-6-(2,2,2-trifluoro-1-hydroxyethylidene)cyclohexanone | 0.36$\pm$0.15 | 0.49$\pm$0.24 | S2 |
| 59 | 1-[1-(3-fluorobenzyl)-1H-benzimidazol-2-yl]-N-[3-(methylthio)phenyl]piperidine-4-carboxamide | 0.45$\pm$0.13 | 0.63$\pm$0.21 | S2 |
| 60 | 4-[3-(1,3-benzodioxol-5-ylamino)imidazo[1,2-a]pyrazin-2-yl]phenol | 1.21$\pm$0.30 | 1.86$\pm$0.20 | S2 |

The relative mRNA expression levels of IL-6 and IL-8 in IL-1b-stimulated GFs vs. IL-1b-stimulated GFs in the presence of chemicals was determined by real- time PCR (n = 3). mRNA expression levels were calculated using the 2^−ΔΔCt^ method, and target gene expression was normalized to that of the GAPDH housekeeping gene. The data are presented as the mean ± SD for triplicates per sample. **p < 0.01; ***p < 0.001.
