# Supplementary material for: DPIE [2-(1,2-diphenyl-1H-indol-3-yl)ethanamine] Augments Pro-Inflammatory Cytokine Production in IL-1β-Stimulated Primary Human Oral Cells
Source: Int J Mol Sci. 2018 Jun 22;19(7):1835. doi: 10.3390/ijms19071835 (PMC6073580; doi:10.3390/ijms19071835)
Supplement: Supplementary file 1 [file ijms-19-01835-s001.zip › ijms-321468 supplementary materials/Supplementary Table 2_sha.docx]

| Supplementary Table 2. Primers used in this study. | | |
| --- | --- | --- |
| Gene | Primer sequence (5’→3’) | |
| GAPDH | Forward | ACCCCTTCATTGACCTCAAC |
|  | Reverse | CTTGACGGTGCCATGGAATT |
| IL-6 | Forward | AGGGCTCTTCGGGAAATGTA |
|  | Reverse | TGCCCAGTGGACAGGTTTC |
| IL-8 | Forward | TTTCTGTTAAATCTGGCAACCCTAGT |
|  | Reverse | ATAAAGGAGAAACCAAGGCACAGT |
| COX-2 | Forward | TCCTTGAAAGGACTTATGGGTAAT |
|  | Reverse | CTGAATGAAGTAAAGGGACAGC |
| iNOS | Forward | GGTGGAAGCGGTAACAAAGG |
|  | Reverse | TGCTTGGTGGCGAAGATGA |
| TNF-α | Forward | GTCACTCATTGCTGAGCCTCT |
|  | Reverse | AGCTTCTTCCCACCCACAAG |
